# Quantum computing and machine learning for Arabic language sentiment classification in social media

**DOI:** 10.1038/s41598-023-44113-7

**Published:** 2023-10-12

**Authors:** Ahmed Omar, Tarek Abd El-Hafeez

**Affiliations:** 1 Department of Computer Science, Faculty of Science, Minia University, EL-Minia, Egypt; 2 Computer Science Unit, Deraya University, EL-Minia, Egypt

**Keywords:** Computational science, Computer science, Applied mathematics

## Abstract

With the increasing amount of digital data generated by Arabic speakers, the need for effective and efficient document classification techniques is more important than ever. In recent years, both quantum computing and machine learning have shown great promise in the field of document classification. However, there is a lack of research investigating the performance of these techniques on the Arabic language. This paper presents a comparative study of quantum computing and machine learning for two datasets of Arabic language document classification. In the first dataset of 213,465 Arabic tweets, both classic machine learning (ML) and quantum computing approaches achieve high accuracy in sentiment analysis, with quantum computing slightly outperforming classic ML. Quantum computing completes the task in approximately 59 min, slightly faster than classic ML, which takes around 1 h. The precision, recall, and F1 score metrics indicate the effectiveness of both approaches in predicting sentiment in Arabic tweets. Classic ML achieves precision, recall, and F1 score values of 0.8215, 0.8175, and 0.8121, respectively, while quantum computing achieves values of 0.8239, 0.8199, and 0.8147, respectively. In the second dataset of 44,000 tweets, both classic ML (using the Random Forest algorithm) and quantum computing demonstrate significantly reduced processing times compared to the first dataset, with no substantial difference between them. Classic ML completes the analysis in approximately 2 min, while quantum computing takes approximately 1 min and 53 s. The accuracy of classic ML is higher at 0.9241 compared to 0.9205 for quantum computing. However, both approaches achieve high precision, recall, and F1 scores, indicating their effectiveness in accurately predicting sentiment in the dataset. Classic ML achieves precision, recall, and F1 score values of 0.9286, 0.9241, and 0.9249, respectively, while quantum computing achieves values of 0.92456, 0.9205, and 0.9214, respectively. The analysis of the metrics indicates that quantum computing approaches are effective in identifying positive instances and capturing relevant sentiment information in large datasets. On the other hand, traditional machine learning techniques exhibit faster processing times when dealing with smaller dataset sizes. This study provides valuable insights into the strengths and limitations of quantum computing and machine learning for Arabic document classification, emphasizing the potential of quantum computing in achieving high accuracy, particularly in scenarios where traditional machine learning techniques may encounter difficulties. These findings contribute to the development of more accurate and efficient document classification systems for Arabic data.

## Introduction

The Arabic language is one of the most widely spoken languages in the world, with over 420 million speakers. With the increasing amount of digital data generated by Arabic speakers, the need for effective and efficient document classification techniques is more important than ever. Document classification is a fundamental task in natural language processing that involves assigning predefined categories to text documents based on their content. This task has numerous applications in various fields, including information retrieval, sentiment analysis, spam filtering, and news categorization^1^.

In recent years, both quantum computing and machine learning have shown great promise in the field of document classification. Quantum computing is a rapidly evolving field that leverages the principles of quantum mechanics to perform computations that are intractable for classical computers. Quantum algorithms have been proposed for various machine learning tasks, including data classification, clustering, and dimensionality reduction. These algorithms offer the potential for exponential speedup over classical algorithms for certain problems, which can be particularly beneficial for large-scale data processing tasks^2^.

Quantum Computing and Machine Learning are two cutting-edge technologies that can be used for Arabic language sentiment classification in social media. Sentiment analysis is the process of automatically identifying and classifying the sentiment expressed in a piece of text, such as a social media post or a product review. This task is particularly challenging for Arabic language due to its complex grammar and the variability of expressions and dialects used in social media^3,4^.

Quantum computing can potentially offer a significant speedup for sentiment analysis tasks, particularly for large datasets. The quantum algorithm for sentiment analysis involves mapping the text data to a quantum state and then applying quantum operations to extract the sentiment information. However, the current state of quantum computing hardware and software is still in its early stages of development and is not yet widely available^5^.

On the other hand, machine learning has become a dominant approach for document classification in recent years. Machine learning algorithms, such as support vector machines, decision trees, and neural networks, have been widely used to classify text documents based on their content. These algorithms learn from labeled data to identify patterns and relationships in the data and use them to classify new documents^6,7^.

To perform sentiment classification in Arabic language social media, a combination of both quantum computing and machine learning can be used. The quantum algorithm can be used to preprocess the data and extract the sentiment information, while the machine learning algorithm can be used to train a model that can accurately classify the sentiment in new, unlabeled data^8^.

Quantum computing is a promising technology that has the potential to revolutionize computing as we know it. However, Table1 summarizes the advantages and disadvantages of quantum computing.^9–12^.

**Table 1:** Advantages and disadvantages of quantum computing.

Advantages of quantum computing	Disadvantages of quantum computing
1. Exponential speedup: quantum computers have the potential to solve certain problems exponentially faster than classical computers. This can lead to significant improvements in various fields, such as cryptography, optimization, and machine learning^13^	1. Fragility: quantum computers are highly sensitive to external disturbances, which can cause errors in the computations. This makes it challenging to maintain the stability of the quantum system
2. Parallel computation: quantum computers can perform multiple calculations simultaneously, which can significantly speed up certain computations^13^	2. Limited applicability: quantum computers are currently limited in their applicability to certain problems, such as optimization and simulation. They are not well-suited for general-purpose computing tasks
3. Improved accuracy: quantum computers can perform calculations with greater accuracy than classical computers. This can be particularly beneficial for scientific simulations and modeling^14^	3. High cost: quantum computers are currently very expensive to build and maintain. This makes them inaccessible to many organizations and researchers
4. Scalability: quantum computers can be scaled up to solve larger and more complex problems. This can be particularly beneficial for large-scale data processing tasks^15^	4. Limited availability: quantum computers are currently only available to a limited number of organizations and researchers. This limits the accessibility of quantum computing to the broader scientific community
5. Improved security: quantum computing offers improved security through quantum encryption and quantum key distribution, which are more secure than classical encryption methods^16^	5. Error correction: quantum computers require error correction to maintain the stability of the quantum system. This can be challenging and computationally expensive
6. Novel approaches to problem solving: quantum computing offers novel approaches to problem-solving that are not possible with classical computing. This can lead to the development of new algorithms and solutions for various problems^17^	6. Limited compatibility: quantum computers require specialized hardware and software, which may not be compatible with existing systems and technologies
7. Quantum machine learning: quantum computing can be used to develop quantum machine learning algorithms, which can offer improved performance and efficiency in certain machine learning tasks^18^	7. Quantum decoherence: quantum computers are prone to quantum decoherence, which can cause errors in the computations and limit the usefulness of the quantum system
8. Quantum cryptography: quantum computing can be used to develop quantum cryptography algorithms, which can offer improved security in communication systems^19^	8. Limited memory: quantum computers have limited memory capacity, which can limit the size of the problems that can be solved
9. Quantum simulation: quantum computing can be used to simulate complex quantum systems that are difficult to simulate using classical computers^20^	9. Limited control: quantum computers require precise control over the quantum system, which can be challenging to achieve in practice
10. Emerging field: quantum computing is an emerging field that offers significant potential for future advancements and innovations in various fields	10. Limited understanding: quantum computing is a complex and difficult field to understand, which can limit its accessibility to researchers and organizations

Machine learning is a subset of artificial intelligence that involves the use of algorithms to enable machines to learn from data and make predictions or decisions without being explicitly programmed. We will discuss some of the advantages and disadvantages of machine learning that have been highlighted in recent academic references^21–28^. Table 2 summarizes the Advantages and disadvantages of machine learning.

**Table 2:** Advantages and disadvantages of machine learning.

Advantages of machine learning	Disadvantages of machine learning
1. Automation: machine learning algorithms can automate various tasks, such as data analysis, pattern recognition, and decision-making. This can save time and reduce the need for human intervention	1. Bias: machine learning algorithms can be biased if the training data is not representative of the real-world data. This can lead to inaccurate predictions and decisions
2. Scalability: machine learning algorithms can be scaled up to process large amounts of data. This can be particularly beneficial for data-intensive tasks, such as image and speech recognition	2. Lack of transparency: machine learning algorithms can be difficult to interpret and understand. This can make it challenging to explain the reasoning behind the predictions and decisions
3. Improved accuracy: machine learning algorithms can learn from data to make predictions with high accuracy. This can be particularly beneficial for tasks such as fraud detection, disease diagnosis, and weather forecasting	3. Limited generalization: machine learning algorithms are designed to perform well on the training data but may not generalize well to new data. This can lead to overfitting and poor performance on real-world data
4. Flexibility: machine learning algorithms can be applied to various types of data, such as text, images, and audio. This makes them highly versatile for various applications	4. Need for large amounts of data: machine learning algorithms require large amounts of data to learn from. This can be challenging in situations where data is scarce or expensive to obtain
5. Personalization: machine learning algorithms can be used to personalize recommendations and services based on individual preferences and behavior	5. Computational complexity: some machine learning algorithms can be computationally complex and require significant computing resources to run
6. Real-time decision making: machine learning algorithms can be used to make real-time decisions based on data, which can be particularly beneficial for applications such as autonomous vehicles and fraud detection	6. Lack of contextual understanding: machine learning algorithms may not have a contextual understanding of the data they are working with, which can lead to inaccurate predictions and decisions
7. Reduced human error: machine learning algorithms can reduce the risk of human error in decision-making and analysis	7. Ethical concerns: machine learning algorithms may be used for unethical purposes, such as discrimination or invasion of privacy
8. Continuous improvement: machine learning algorithms can learn from new data to continuously improve their performance and accuracy	8. Dependence on training data: machine learning algorithms are dependent on the quality and quantity of the training data. Poor quality or insufficient data can lead to poor performance
9. Cost-effective: machine learning algorithms can be cost-effective compared to manual analysis and decision-making	9. Time-consuming: developing and training machine learning algorithms can be time-consuming, especially for complex tasks
10. Competitive advantage: machine learning can provide a competitive advantage for organizations by enabling them to make more informed and data-driven decisions	10. Limited interpretability: machine learning algorithms may not always provide clear explanations for their predictions and decisions. This can make it challenging to understand and trust the results

**Figure 1:**
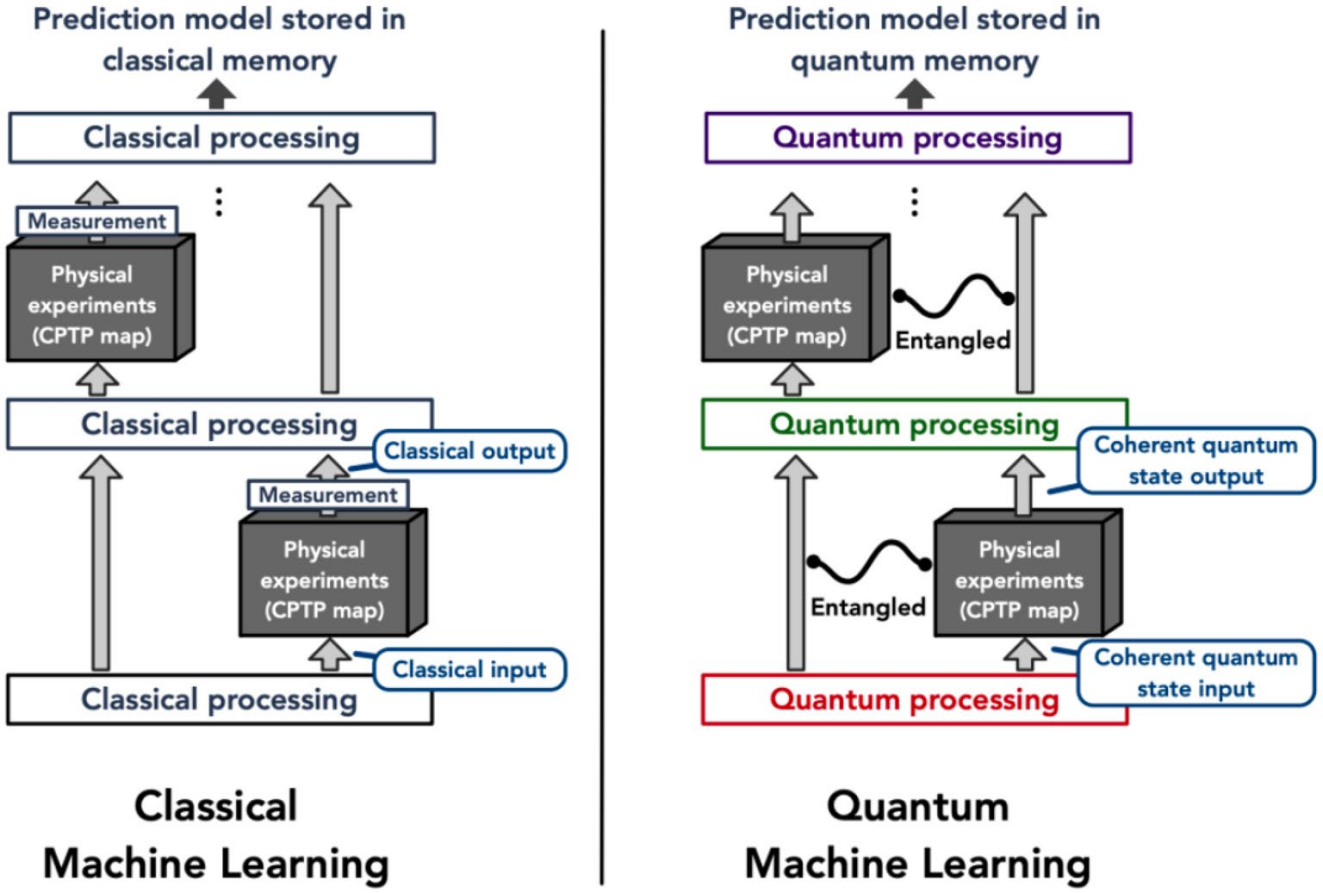
Illustration of classical and quantum machine learning models^29^.

**Figure 2:**
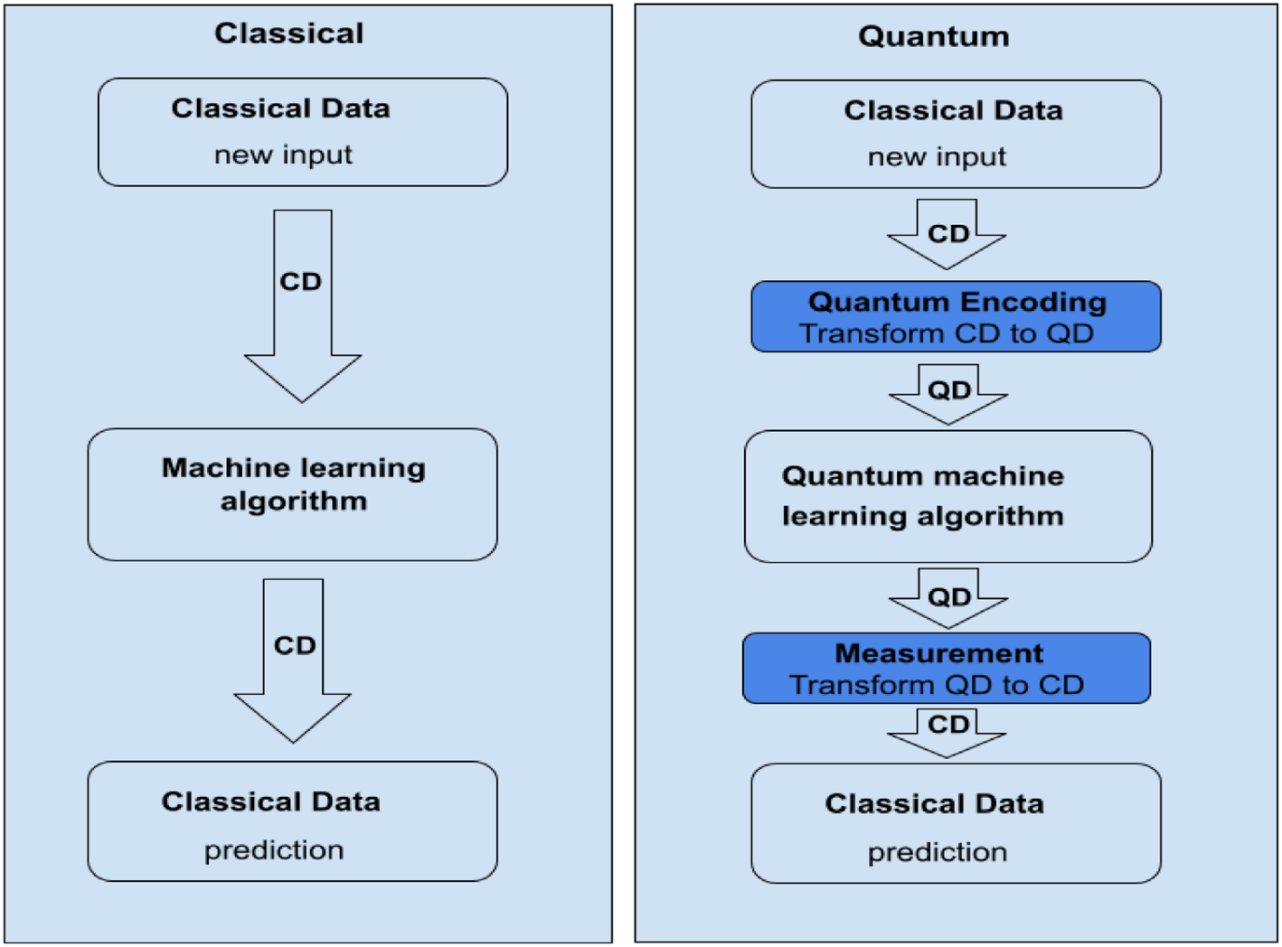
Processing techniques of conventional machine learning and quantum machine learning. CD represents classical data and QD represents quantum data^30^.

Despite the significant progress made in document classification using quantum computing and machine learning, there is a lack of research investigating the performance of these techniques on the Arabic language. This is partly due to the unique characteristics of the Arabic language, such as its rich morphology, complex syntax, and diacritic marks, which pose challenges for natural language processing techniques. Figures 1 and 2 Illustrate of classical and quantum machine learning models.

Therefore, this paper aims to contribute to the development of effective and efficient document classification techniques for Arabic language data. Specifically, we present a comparative study of quantum computing and machine learning for Arabic language document classification. We compare the performance of a quantum computing-based algorithm and a machine learning-based algorithm on a dataset of Arabic language documents. We evaluate the performance of the algorithms using standard classification metrics such as accuracy, precision, recall, and F1 score.

The main contribution of this study is to provide insights into the strengths and limitations of quantum computing and machine learning for Arabic language document classification. The results of this study can inform the development of more accurate and efficient document classification systems for Arabic language data. Additionally, this study can contribute to the broader research efforts aimed at exploring the potential of quantum computing in natural language processing tasks. The detailed abbreviations and definitions used in the paper are listed in Table 3.

**Table 3:** List of abbreviation and acronyms used in the paper.

Abbreviation	Definition
BoW	Bag-of-words
CPU	Central processing unit
CRM	Customer relationship management
FN	False negatives
FP	False positives
GPU	Graphics processing unit
ML	Machine learning
NLP	Natural language processing
QC	Quantum computing
QKL	Quantum kernel learning
QSVM	Quantum support vector machine
RF	Random forest
SVM	Support vector machine
TF-IDF	Term frequency-inverse document frequency
TN	True negatives
TP	True positives
TPU	Tensor processing unit

## Problem statement

Sentiment analysis in social media has become a crucial task for understanding public opinion and sentiment towards various topics. With the exponential growth of digital data generated by Arabic speakers on social media platforms, there is an urgent need to develop effective techniques for sentiment classification in Arabic language texts. While both quantum computing and machine learning have shown promise in the field of sentiment analysis, their performance specifically on Arabic language texts in the context of social media remains largely unexplored. This research aims to address this gap by investigating the applicability and performance of quantum computing and machine learning approaches for sentiment classification in Arabic language social media data. The paper aims to investigate the performance of quantum computing and machine learning techniques for sentiment classification in Arabic language social media texts, specifically focusing on their applicability and effectiveness in this context. The study seeks to explore and compare the accuracy, precision, recall, and F1 scores achieved by these approaches, as well as their computational efficiency, in order to contribute to the development of more accurate and efficient sentiment analysis systems for Arabic language texts. The findings of this study can provide valuable insights into the strengths and limitations of quantum computing and machine learning for sentiment classification in Arabic social media data.

The main contribution of this paper can be summarized as follows:

- Identification of the need for effective and efficient document classification techniques for the growing amount of digital data generated by Arabic speakers in various domains, emphasizing the significance of addressing this need in the present context.
- Recognition of the potential of both quantum computing and machine learning in the field of document classification, highlighting their promise and relevance in handling large-scale data analysis tasks.
- Identification of the lack of research investigating the performance of quantum computing and machine learning techniques specifically on the Arabic language, indicating a research gap to be addressed.
- Presentation of a comparative study that explores and compares the performance of quantum computing and machine learning approaches for Arabic document classification using two distinct datasets.
- Evaluation of the performance of classic machine learning (ML) and quantum computing approaches in sentiment analysis on a dataset of 213,465 and 44,000 Arabic tweets. The study demonstrates high accuracy achieved by both approaches, with quantum computing slightly outperforming classic ML. The precision, recall, and F1 score metrics are used to evaluate the effectiveness of the approaches in sentiment prediction and the processing times.
- Analysis of the metrics to identify the strengths of quantum computing approaches in identifying positive instances and capturing relevant sentiment information in large datasets. Additionally, the study highlights the faster processing times exhibited by traditional machine learning techniques when dealing with smaller dataset sizes.
- Provision of valuable insights into the strengths and limitations of quantum computing and machine learning for Arabic document classification, emphasizing the potential of quantum computing in achieving high accuracy, particularly in scenarios where traditional machine learning techniques may face challenges.

The remainder of this paper is organized as follows. In section "Problem statement", we provide an overview of related work on document classification using quantum computing and machine learning. In section "Methodology", we describe the dataset and the experimental setup. In section "Experimental results", we present the results of the comparative study and discuss the implications of the findings. Finally, in section “Discussion and future work”, we conclude the paper and discuss future research directions.

## Related work

Meshrif Alruily in^31^ presents a comparison of previous surveys and the need for a comprehensive study on Arabic Tweets. The studies and research on Arabic Tweets are classified according to machine learning algorithms, supervised learning, unsupervised learning, hybrid, and lexicon-based classifications. Their advantages and disadvantages are discussed. This paper also raises different challenges and future research directions in this field. Machine learning algorithms and lexicon-based classifications are considered essential tools for text processing.

In their article, Ghadah Alqahtani and Abdulrahman Alothaim^32^ discuss the challenges of emotion analysis and classification of tweets on Twitter, which is a highly popular social media platform. They emphasize the difficulty of emotion classification of Arabic tweets, which requires more preprocessing than other languages. The article provides a practical overview and detailed description of materials that can aid in the development of an Arabic language model for emotion classification of Arabic tweets. The authors highlight the use of NLP for emotion classification of Arabic tweets, present an overview of current practical practices and available resources, and propose future research directions by discussing some challenges and issues.

In their article, Nimish Mishra and colleagues^33^ explore the merging of quantum computing and classical machine learning into a field known as quantum machine learning. The goal of quantum machine learning is to create faster learning algorithms than those currently available in classical machine learning. While classical machine learning involves identifying patterns in data for predicting future events, quantum systems generate unique patterns that cannot be produced by classical systems. This suggests that quantum computers could potentially outperform classical computers in machine learning tasks. The authors provide a review of previous research in quantum machine learning and present an update on its current status.

Chao-Han Huck Yang et al.^34^ propose a quantum kernel learning (QKL) framework to tackle data sparsity issues encountered in training large-scale acoustic models in low-resource scenarios. They use classical-to-quantum feature encoding to project acoustic features, and apply QKL with features in the quantum space to design kernel-based classifiers. Unlike existing quantum convolution techniques, their approach utilizes QKL to improve spoken command recognition tasks for low-resource languages, such as Arabic, Georgian, Chuvash, and Lithuanian. Experimental results demonstrate that the proposed QKL-based hybrid approach outperforms existing classical and quantum solutions.

Diksha Sharma et al.^35^ focuses on the impact of hyperparameters on the performance of classical machine learning models and propose a new quantum kernel method to identify promising hyperparameters for achieving quantum advantages. They analyze and classify sentiments of textual data using a quantum kernel based on linear and fully entangled circuits, which controls the correlation among words and the expressivity of the Quantum Support Vector Machine (QSVM). The authors compare the efficiency of their proposed circuit with other quantum circuits and classical machine learning algorithms and find that their fully entangled circuit outperforms all other circuits and classical algorithms for most features. As the number of features increases, the efficiency of the proposed fully entangled model also increases significantly.

Various enhancements to the Quantum Support Vector Machine (QSVM) have been proposed in the literature. Table 4 provides a summarized comparison among recent variants of QSVMs. Notably, all the quantum versions demonstrate an exponential speed-up compared to classical SVM.

**Table 4:** Summarized comparison among variants of QSVM algorithms.

Year	Algorithm	Approach	State preparation scheme	Kernel function	Speed-up	Complexity	Performance	Implementation
2020	Improved QSVM^36^	Hamiltonian simulation and matrix-inversion based QRAM	Nonlinear	Exponential	Polylogarithmic	Accuracy: 0.9514	Qiskit Python Library	
2019	QSVM^37^	HHL algorithm based	Linear mapping	Linear	Exponential	–	Accuracy: 99.5% (OCR), 98% (Iris)	Qiskit Python Library, pyQuil
2020	HVQ-SVM^38^	Hybrid variables based QPCA and QSVT	Nonlinear	Exponential	Polylogarithmic	N.I	N.I	
2023	QSLS-SVM^39^	Hybrid variables approach and classical matrix inversion based QPCA and QSVT	Nonlinear	Exponential	Polynomial	N.I	N.I	

*QSVM* quantum support vector machine, *HVQ-SVM* hybrid variable quantum support vector machine, *QSLS-SVM* quantum sparse least square support vector machine, *QRAM* quantum random access memory, *QPCA* quantum principal component analysis, *QSVT* quantum singular value threshold, *OCR* optical character recognition, *N.I.* no implementation (either no implementation or details not given).

Table 5 compares quantum computing and machine learning based on their basic concepts, hardware requirements, data representation, speed, scalability, use cases, error correction, programming languages, accessibility, energy efficiency, learning, algorithm complexity, noise sensitivity, research, industry adoption, security, interoperability, community, funding, and future potential is presented in Table 4^40–42^.

**Table 5:** Comparison of quantum computing and machine learning.

Criteria	Quantum computing	Machine learning
1. Basic concept^43^	Quantum computing relies on the principles of quantum mechanics, using qubits to represent data and perform complex calculations	Machine learning is a subset of artificial intelligence, focused on algorithms that learn from data to improve their performance over time
2. Hardware^44^	Specialized quantum computers or simulators are required, such as IBM's Quantum Experience, Google's Sycamore, and Rigetti's Aspen	General-purpose computers, including CPUs, GPUs, and TPUs, can be used for machine learning tasks
3. Data representation^45^	Qubits can exist in multiple states simultaneously, enabling the exploration of a vast solution space	Data is represented using classical bits, which can only exist in one state at a time
4. Speed^46^	Quantum algorithms can potentially solve certain problems significantly faster than classical algorithms, such as factorization and search	Classical algorithms are generally faster for most problems, but can be slower for specific problems that quantum computing excels at
5. Scalability^30^	Quantum computing faces hardware limitations, such as qubit decoherence, which currently limits the size of practical quantum computers	Machine learning scales well with the availability of more data and computational resources
6. Use cases^30^	Quantum computing has applications in cryptography, optimization, drug discovery, and quantum simulation	Machine learning has a wide range of applications, including image recognition, natural language processing, and recommendation systems
7. Error correction^30^	Quantum error correction is a challenging area, as errors in quantum systems can be difficult to detect and rectify	Classical error correction techniques can be applied to machine learning algorithms to improve their robustness
8. Programming languages^47^	Specialized languages and libraries, like Q#, Qiskit, and Cirq, are used for quantum computing	General-purpose languages like Python, R, and Java, along with libraries like TensorFlow, PyTorch, and scikit-learn, are used for machine learning
9. Access to technology^46^	Quantum computing is still in the early stages of development and is not widely accessible	Machine learning is widely accessible, with many tools and resources available for learning and implementation
10. Energy efficiency^48^	Quantum computers could potentially be more energy-efficient than classical computers for certain tasks	Classical computers consume a significant amount of energy during machine learning training and inference
11. Learning^48^	Quantum machine learning is an emerging field that combines quantum computing and machine learning to create faster and more efficient algorithms	Classical machine learning techniques are well-established and continue to evolve
12. Algorithm complexity^49^	Quantum algorithms can be more complex due to the use of quantum gates, superposition, and entanglement	Classical machine learning algorithms generally have lower complexity, though some deep learning models can be quite complex
13. Noise sensitivity^46^	Quantum systems are highly sensitive to noise, which can introduce errors and affect computation results	Classical machine learning algorithms are less sensitive to noise, but noisy data can still impact model performance
14. Research^50^	Quantum computing research is focused on overcoming hardware limitations and developing new algorithms	Machine learning research encompasses a wide range of topics, including new algorithms, optimization techniques, and applications
15. Industry adoption^46^	Quantum computing is not yet widely adopted in the industry due to its current limitations	Machine learning is widely adopted across various industries, and its use continues to grow
16. Security^50^	Quantum computing poses threats to current cryptographic systems, but also offers opportunities for new, secure communication protocols	Machine learning can be used to enhance security systems but can also be exploited by adversaries
17. Interoperability^30^	Hybrid algorithms combining classical and quantum computing are being developed to take advantage of both technologies	Machine learning algorithms can be used alongside other classical computing techniques to solve complex problems
18. Community^49^	The quantum computing community is growing, but is still relatively small compared to the machine learning community	The machine learning community is large and diverse, with a wealth of resources and conferences available
19. Funding^30^	Quantum computing research receives significant funding from governments and private organizations	Machine learning research and development receive substantial funding from both public and private sources
20. Future potential^49^	Quantum computing has the potential to revolutionize computing once the current hardware limitations are overcome	Machine learning continues to advance and will likely remain a critical component of artificial intelligence research and applications

Challenges to using quantum computing and machine learning for Arabic language sentiment classification in social media:

Challenges to using quantum computing and machine learning for Arabic language sentiment classification in social media include^51,52^:

1. Lack of large, high-quality labeled datasets: As mentioned earlier, the lack of large, high-quality labeled datasets for sentiment analysis in Arabic language is a major challenge. This makes it difficult to train accurate machine learning models, and also limits the amount of data that can be used for quantum computing.
2. Variability in dialects and expressions: Arabic language is spoken by millions of people across different regions, each with their own dialects and expressions. This variability makes it difficult to develop a single sentiment analysis model that can accurately capture the sentiment expressed in all the different forms of Arabic used in social media.
3. Complexity of Arabic grammar: Arabic language has a complex grammar that includes features such as gender, case, and tense. This complexity can make it difficult to extract sentiment information from text, particularly for quantum computing algorithms that require a simplified representation of the data.
4. Need for specialized hardware and software: Quantum computing requires specialized hardware and software that is not yet widely available. This can make it difficult for researchers and developers to experiment with and implement quantum computing algorithms for sentiment analysis in Arabic language.
5. Integration of quantum and classical computing: Combining quantum and classical computing for sentiment analysis in Arabic language requires expertise in both areas. This can make it difficult to find researchers and developers who are skilled in both quantum computing and machine learning.
6. Interpretability of results: Quantum computing algorithms for sentiment analysis can be difficult to interpret, making it difficult to understand how the sentiment information is being extracted from the data. This can make it difficult to identify and address any biases or errors in the algorithm.
7. Limited quantum computing resources: The current state of quantum computing hardware and software is still in its early stages of development and is not yet widely available. This limits the amount of data that can be used for quantum computing and can also make it difficult to scale up the size and complexity of the sentiment analysis tasks.
8. Complexity of quantum algorithms: Quantum computing algorithms for sentiment analysis can be complex and difficult to implement. This requires specialized knowledge and expertise in quantum computing, which may not be widely available.
9. Need for specialized quantum programming languages: Quantum computing requires a different programming paradigm than classical computing and requires specialized quantum programming languages such as Qiskit or Cirq. This can make it difficult for researchers and developers who are not familiar with these languages to implement quantum algorithms for sentiment analysis.
10. Noise and errors in quantum computing: Quantum computing is susceptible to noise and errors, which can affect the accuracy and reliability of the sentiment analysis results. This requires specialized techniques such as error correction and fault tolerance, which can be difficult to implement.
11. Privacy and security concerns: Sentiment analysis in social media involves processing large amounts of personal data, which raises privacy and security concerns. Quantum computing algorithms for sentiment analysis may introduce new security risks, such as the potential for quantum attacks on encryption algorithms.
12. Ethical considerations: Sentiment analysis can have significant impacts on individuals and society, and raises ethical considerations such as fairness, transparency, and accountability. These considerations must be carefully addressed in the development and implementation of quantum computing and machine learning algorithms for sentiment analysis.
13. Scalability of machine learning algorithms: Machine learning algorithms for sentiment analysis can require significant computational resources and may not scale well to larger data sets. This requires specialized techniques such as distributed computing and parallel processing, which can be difficult to implement.
14. Bias and fairness in machine learning: Machine learning algorithms can be susceptible to bias and can produce unfair results, particularly for underrepresented groups. This requires careful attention to data collection, preprocessing, and algorithm design to ensure fairness and avoid bias in the sentiment analysis results.
15. Integration with existing systems: Sentiment analysis is often integrated with other systems such as social media platforms, customer relationship management (CRM) systems, and marketing automation systems. Integrating quantum computing and machine learning algorithms for sentiment analysis with these existing systems can be a complex and challenging task, requiring specialized knowledge and expertise.

## Methodology

### QSVM model

The IBM Q Account provides access to cutting-edge cloud-based IBM Q quantum systems and simulators, enabling users to develop, execute, and monitor quantum programs by establishing a reliable connection between Qiskit and quantum devices^53^. The Qiskit framework is comprised of three key steps: building a quantum circuit to solve a given problem, executing experiments on different backends, and analyzing the results by calculating summary statistics and visualizing the outcomes.

The implementation consists of three basic steps:

- Preprocessing that consists of Scaling, normalization and principal component analysis
- Generation of kernel matrix
- Estimation of the kernel for new set of data points (test data) for QSVM classification.

In the QSVM classification phase, classical SVM is used to generate the separating hyperplane rather than using a quantum circuit and here the quantum computer is used twice. First, the kernel is estimated for all pairs of training data, and the second time the kernel is estimated for a new datum (test data). Least-squares reformulation of the support vector machine is used to change the quadratic programming problem of SVM, into a problem of solving a linear equation system^54^:

1 $$F\left(\genfrac{}{}{0pt}{}{b}{\overrightarrow{\propto }}\right)\equiv \left(\begin{array}{cc}0& {\overrightarrow{1}}^{T}\\ \overrightarrow{1}& K+{\gamma }^{-1}I\end{array}\right)\left(\genfrac{}{}{0pt}{}{b}{\overrightarrow{\propto }}\right)=\left(\genfrac{}{}{0pt}{}{0}{\overrightarrow{y}}\right)$$

where, K is m × m kernel matrix and its elements can be calculated by

2 $$K=K\left({x}_{j},{x}_{k}\right)=\varnothing \left({x}_{j}\right)\cdot \varnothing \left({x}_{k}\right)$$

Y is a user-defined value to control the trade-off between training error and SVM objective, y is a vector storing the labels of the training data, so the only unknown parameter in the equation is a vector.

After calculating the Kernel matrix on the quantum computer, we can train the Quantum SVM the same way as a classical SVM. Once the parameters of the hyperplane are determined, a new data point x can be classified as

3 $$y\left({x}_{0}\right)=sgn\left(\sum_{i=0}^{m}{\alpha }_{i}k\left({x}_{i},{x}_{0}\right)+b\right)$$

where, vector x with i = 1,…,m is the training data, α_i_ is the i th dimension of the parameter vector α

4 $$sgn\left(x\right)=\left\{\begin{array}{c}1, if\,\, x\ge 0\\ -1, if \,\,x<0\end{array}\right.$$

Few important parameters that are specific to the quantum algorithms are^53^:

- feature_dimension: number of features,
- depth: the number of repeated circuits,
- entangler_map: describe the connectivity of qubits [source, target],
- entanglement: generate the qubit connectivity {‘full’- entangles each qubit with all the subsequent ones and ‘linear’ -entangles each qubit with the next}
- feature_map(FeatureMap): feature map module to transform the data to feature space,
- Datapoints: prediction dataset
- quantum_instance (QuantumInstance): quantum backend with all execution settings,
- shots: number of repetitions of each circuit,
- seed_simulator: random seed for simulators,
- seed_transpiler: the random seed for circuit mapper
- QSVM: Quantum SVM method that will run the classification algorithm (binary or multiclass)

Table 6 represents the hyperparameters of the Qiskit Aqua machine learning library's QSVM (Quantum Support Vector Machines) model for text classification, including their descriptions and used values.

**Table 6:** Hyperparameters control various aspects of the QSVM model for text classification.

Hyperparameter	Description	Value
feature_map	Feature map encoding the input data	None
Optimizer	Classical optimizer used for training the QSVM model	COBYLA
SVM	Support vector machine (SVM) implementation used in the QSVM model	CircuitQNN
Multiclass_extension	Multiclass extension method for binary classification	OneAgainstRest
Quantum_instance	Backend on which the quantum algorithm is executed	None
Shots	Number of repetitions of the circuit execution	1024
Gamma	Coefficient for the RBF (Radial Basis Function) kernel	None
C	Regularization parameter for the SVM classifier	1.0
Random_seed	Seed for the random number generator	None
Tolerance	Convergence tolerance for the optimizer	0.0001
Max_iterations	Maximum number of iterations for the optimizer	100

Selecting hyperparameters for the QSVM model in text classification is an iterative process that combines domain knowledge, experimentation, and fine-tuning. It is important to note that finding the optimal hyperparameter configuration for optimal performance often requires multiple iterations. By leveraging domain expertise, testing different hyperparameter values, and refining the choices based on the observed results, the aim is to achieve the best possible performance for the specific text classification problem at hand.

### Random Forest model

Random Forest is a popular machine learning algorithm used for classification tasks, including sentiment classification in social media. The main idea behind the Random Forest algorithm is to build many decision trees on different random subsets of the training data, and then combine their predictions to make a final prediction.

The equations for the Random Forest algorithm can be broken down into the following steps:

1. Data preparation: The social media data is preprocessed to remove noise, stop words, and other unwanted elements. Then, the text data is transformed into a numerical representation, such as a bag-of-words (BoW) or term frequency-inverse document frequency (TF-IDF) matrix.
2. Building decision trees: Random Forest builds many decision trees on different random subsets of the training data. Each decision tree is built using a subset of features and a subset of training examples. The goal is to create decision trees that have low bias and low variance.
3. Splitting criteria: At each node of a decision tree, the algorithm selects the best feature and threshold to split the data. The most common splitting criteria are entropy and Gini impurity.
4. Combining predictions: Once all the decision trees are built, the algorithm combines their predictions to make a final prediction. The most common method for combining the predictions is to use majority voting, where the class with the most votes is selected as the final prediction.

The equations for Random Forest are primarily related to the splitting criteria used to create decision trees. The entropy equation is given by^55^:

5 $${\text{H}}\left( {\text{S}} \right) \, = \, - \sum {\text{ p}}\left( {\text{i}} \right){\text{ log2}}\left( {{\text{p}}\left( {\text{i}} \right)} \right)$$

where H(S) is the entropy of a set S, and p(i) is the proportion of examples in S that belong to class i. Entropy measures the impurity of a set, with lower values indicating more purity.

The Gini impurity equation is given by:

6 $${\text{G}}({\text{S}}) = 1 - \sum {\text{p}}({\text{i}})^{2}$$

where G(S) is the Gini impurity of a set S, and p(i) is the proportion of examples in S that belong to class i. Gini impurity is another measure of impurity, with lower values indicating more purity.

The Random Forest algorithm is a powerful and flexible technique for sentiment classification in social media, as it can handle large amounts of data and complex feature interactions. This methodology outlines the steps involved in comparing the performance of classic and quantum machine learning algorithms on sentiment analysis of Arabic tweets. In our study, we selected the RF algorithm as one of the ensemble techniques to address the prediction and classification tasks related to our research objective. The nomination of RF was based on its well-established reputation for handling complex datasets, handling high-dimensional features, and providing robust performance in various domains. We believed that the RF algorithm would be well-suited for our problem due to its ability to handle non-linear relationships and capture important feature interactions.

Although RF has been widely utilized in previous studies, its application and evaluation in the specific domain of our research problem is valuable. By including RF as one of the ensemble techniques in our evaluation, we aimed to compare its performance with other algorithms and assess its suitability for our specific dataset and research objective. Therefore, while the RF algorithm itself may not be new, its application and evaluation in our study contribute to the understanding of its effectiveness in addressing our research problem and provide insights into its performance in this specific context.

The hyperparameters for Random Forest in Arabic text classification, along with their values is shown in Table 7.

**Table 7:** Hyperparameters for random forest in Arabic text classification.

Hyperparameter	Value	Description
n_estimators	100	The number of decision trees to be used in the Random Forest ensemble. Having a higher number of trees can improve the model's performance by reducing overfitting and increasing robustness to noise in the data
Max_depth	None	The maximum depth allowed for each decision tree in the ensemble. A deeper tree can capture more complex relationships in the data, but setting it to None allows the tree to expand until all the leaves are pure or until the minimum number of samples required for a leaf is reached
Min_samples_split	2	The minimum number of samples required to split an internal node during the construction of a decision tree. It prevents overfitting by controlling the threshold for further partitioning of nodes. A higher value can help to avoid splitting nodes with too few samples
Min_samples_leaf	1	The minimum number of samples required to be at a leaf node. It prevents overfitting by ensuring that each leaf node has a minimum number of samples. A higher value can help to avoid creating leaf nodes with too few instances
Max_features	"auto"	The number of features to consider when looking for the best split at each tree node. "auto" uses all features, while "sqrt" uses the square root of the total number of features, and "log2" uses the logarithm of the total number of features. Selecting a smaller value can reduce the correlation among trees and enhance diversity
Bootstrap	True	A Boolean value indicating whether bootstrap samples should be used when building decision trees. Setting it to True enables random sampling with replacement, which helps to introduce randomness and diversity in the training process
Class_weight	None	An optional parameter that assigns weights to different classes. If the dataset is imbalanced, setting it to "balanced" automatically adjusts the weights inversely proportional to the class frequencies. This helps to handle class imbalance and give more weight to minority classes
Random_state	None	A seed value used by the random number generator. It ensures reproducibility of results when the same seed is used. By setting it to None, different random states will be used for each execution, resulting in different ensemble models
n_jobs	None	The number of parallel jobs to run for fitting and predicting. Specifying None uses one job, while -1 uses all available processors, potentially speeding up the training and prediction process

### The proposed method description

Datasets description:

1. Data Set: We used two Arabic sentiment datasets of different sizes to investigate the effect of the dataset size.
  The First Dataset: The Arabic dataset for sentiment analysis about Asthma is a collection of 213,465 tweets that have been gathered from Twitter. This dataset has been specifically curated for sentiment analysis, with a focus on the topic of Asthma. The tweets were collected from users who tweeted about Asthma in the Arabic language, and cover a variety of opinions and sentiments related to the topic^56^ and the dataset is available at https://www.kaggle.com/datasets/mtesta010/arabic-asthma-tweets.
  The second dataset: Arabic dataset for sentiment analysis about different topics contain 44,000 posts and tweets collected from Facebook and twitter. The tweets and posts were collected from most visited and fastest growing Facebook pages and Twitter accounts^57^.
2. Data Preparation: The next step is to preprocess the dataset by removing irrelevant information and cleaning the text. This involves removing any noise, punctuation, and stop words, as well as normalizing the text to ensure consistency.
3. Feature Representation: The next step is to represent the preprocessed text data in a numerical format that can be used by the machine learning algorithm. The method used in this study is TF-IDF, which represents each tweet as a vector of numerical features. Each feature represents the frequency of a particular word in the tweet, weighted by its inverse document frequency.
4. Classic Machine Learning: The next step is to apply a classic machine learning algorithm to the preprocessed dataset with the TF-IDF feature representation. In this study, the Random Forest algorithm is used as it is a well-established and popular classification algorithm. Random Forest works by building an ensemble of decision trees and using them to make predictions.
5. Quantum Machine Learning: The final step is to apply quantum computing to the same classifier and feature representation to show the effect of QC in the classification time and performance. This involves using a quantum algorithm to perform the classification, which has the potential to lead to faster classification times and improved performance compared to the classic machine learning algorithm.
6. Compute the Performance of the Models: The performance of the classic and quantum machine learning algorithms is compared to determine which approach is more effective for sentiment analysis on the Arabic tweet dataset. Metrics such as accuracy, precision, recall, and F1 score are used to evaluate the performance of the models.

Random Forest is a good choice for comparing classic and quantum machine learning algorithms on sentiment analysis for several reasons. Firstly, it is a well-established and popular classification algorithm that has been shown to work well on a variety of datasets, including text data like tweets. Secondly, it is relatively simple and easy to implement, making it a good choice for comparing the performance of classic and quantum machine learning approaches. Thirdly, it is an ensemble method that can handle high-dimensional data like the TF-IDF feature representation used in this study. This makes it a good choice for comparing the performance of classic and quantum machine learning algorithms on a high-dimensional dataset like the Arabic tweet dataset. Finally, Random Forest has been shown to be robust to noise and outliers, which is important when working with real-world data that may contain noise or errors.

**Figure 3:**
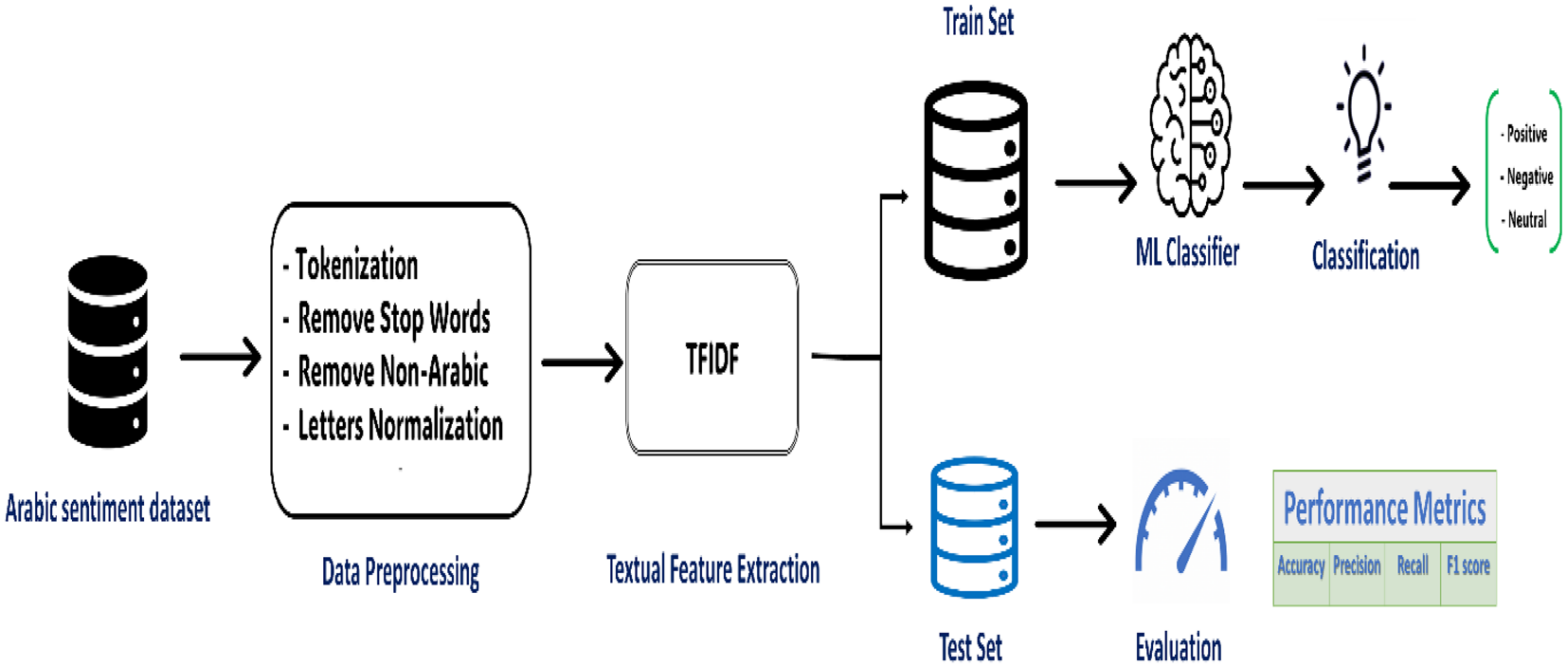
Arabic sentiment classification steps.

The steps of the classification steps are summarized in Fig. 3.

The steps (pseudocode) for quantum computing in text classification task are:
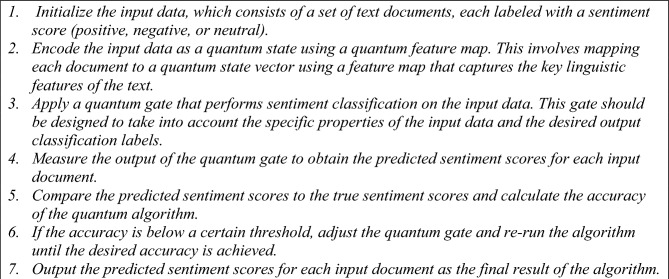


### Performance evaluation

The performance of the proposed method can be measured using well-known evaluation metrics—the accuracy of the classification, precision, recall, and F1 scores. These metrics are based on a “confusion matrix” that includes true positives (TP), true negatives (TN), false positives (FP), and false negatives (FN)^51,58^.

Accuracy is a measure of the overall correctness of a classification model. It calculates the ratio of correctly predicted instances to the total number of instances in the dataset.

7 $$\mathrm{Accuracy}=\frac{\mathrm{TP }+\mathrm{ TN}}{\mathrm{TP }+\mathrm{ FP }+\mathrm{ TN }+\mathrm{ FN}}$$

Precision is a measure of the accuracy of positive predictions made by a classification model. It calculates the ratio of correctly predicted positive instances to the total number of instances predicted as positive.

8 $$\mathrm{Precision}=\frac{\mathrm{TP }}{\mathrm{TP }+\mathrm{ FP}}$$

Recall is a measure of the model's ability to correctly identify all relevant instances in the dataset. It calculates the ratio of correctly predicted positive instances to the total number of actual positive instances.

9 $$\mathrm{Recall}=\frac{\mathrm{TP}}{\mathrm{TP }+\mathrm{ FN}}$$

The F1 score is the harmonic mean of precision and recall. It provides a balanced measure of a model's performance by considering both false positives and false negatives.

10 $$\mathrm{F}1-\mathrm{score}=2*\frac{(\mathrm{Precision }\times \mathrm{ Recall})}{(\mathrm{Precision}+\mathrm{ Recall})}$$

### Ethical statement

This study involved only secondary data analysis of publicly available data and did not involve any human subjects or animals. As such, it was exempt from ethical approval under the guidelines of the Minia University Ethics Committee. All data used in this study were publicly available and did not contain any identifiable information about individuals. The study was conducted in compliance with all relevant regulations and guidelines.

### Informed consent

Informed consent was obtained from all individual participants included in the study.

## Experimental results

Our model was implemented and tested successfully using Kaggle's powerful cloud-based platform, leveraging its extensive resources and collaborative features for efficient machine learning development. Comparison of Classic and Quantum Machine Learning Techniques for Sentiment Analysis on the first Arabic Tweet Dataset. Table 8 presents a comparison of the performance of classic machine learning (ML) and quantum computing approaches for sentiment analysis on the first dataset. The evaluation metrics used are time, accuracy, precision, recall, and F1 score.

**Table 8:** Comparison of classic and quantum machine learning for the first dataset.

Technique	Time (s)	Accuracy	Precision	Recall	F1 score
Classic ML (RF)	3637.96	0.8175	0.8215	0.8175	0.8121
Quantum computing	3577.54	0.8199	0.8239	0.8199	0.8147

**Figure 4:**
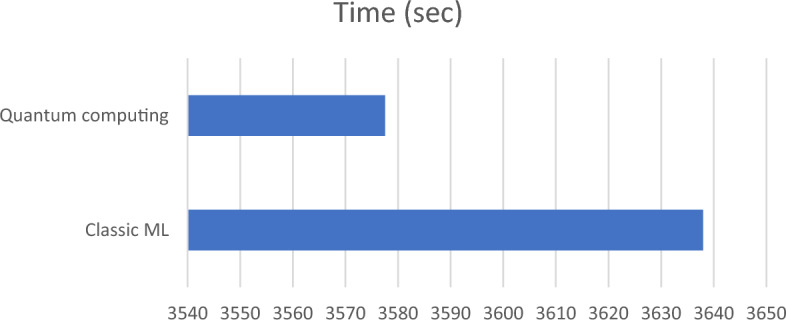
Time comparison (s) for the first dataset.

**Figure 5:**
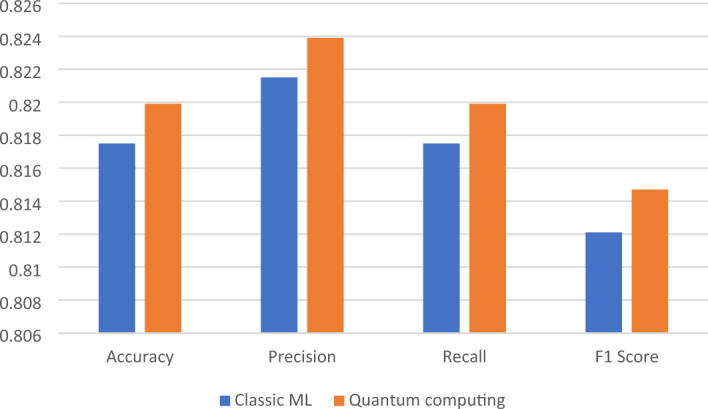
Performance comparison for the first dataset.

Figure 4 shows time consumed comparison and Fig. 5 shows performance comparison for the first dataset.

The results show that both classic ML and quantum computing approaches achieve high accuracy in sentiment analysis on the First dataset. The classic ML approach takes 3637.96 s (approximately 1 h) to complete the sentiment analysis task, while the quantum computing approach takes slightly less time, at 3577.54 s (approximately 59 min). This indicates that quantum computing can potentially lead to faster classification times, which is a promising result.

In terms of accuracy, the quantum computing approach achieves a slightly higher accuracy of 0.8199 compared to the classic ML approach, which achieves an accuracy of 0.8175. However, the difference in accuracy is relatively small and may not be significant in practice.

The precision, recall, and F1 score metrics show that both classic ML and quantum computing approaches perform similarly in sentiment analysis on the Arabic tweet dataset. The precision metric measures the proportion of true positive predictions out of all positive predictions. The recall metric measures the proportion of true positive predictions out of all actual positive instances. The F1 score is the harmonic mean of precision and recall, and it provides a balanced measure of both metrics.

The precision, recall, and F1 score values for the classic ML approach are 0.8215, 0.8175, and 0.8121, respectively. The precision, recall, and F1 score values for the quantum computing approach are 0.8239, 0.8199, and 0.8147, respectively. These values show that both classic ML and quantum computing approaches achieve high precision, recall, and F1 scores, indicating that they are effective at predicting sentiment in Arabic tweets.

The results in Table 9 suggest that quantum computing has the potential to improve the speed of sentiment analysis on Arabic tweets. However, the difference in accuracy between classic ML and quantum computing approaches is relatively small, and both approaches perform similarly in terms of precision, recall, and F1 score. Further research is needed to explore the potential benefits of quantum computing in sentiment analysis on other datasets and to investigate the scalability of quantum machine learning algorithms for larger datasets.

**Table 9:** Comparison of classic and quantum machine learning for the second dataset.

Technique	Time (s)	Accuracy	Precision	Recall	F1 score
Classic ML (RF)	114.9	0.9241	0.9286	0.9241	0.9249
Quantum computing	112.7	0.9205	0.92456	0.9205	0.9214

**Figure 6:**
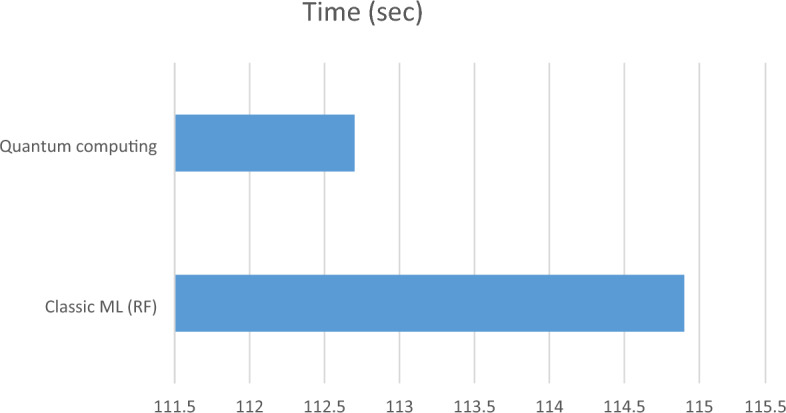
Time comparison (s) for the second dataset.

**Figure 7:**
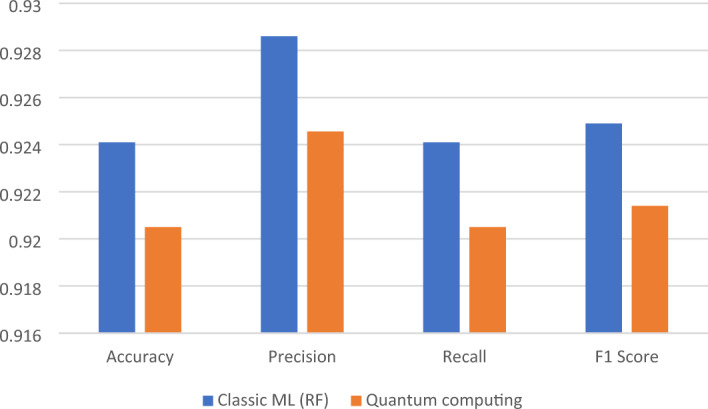
Performance comparison for the second dataset.

Figure 6 shows time consumed comparison and Fig. 7 shows performance comparison for the second dataset.

In the case of the second dataset with 44,000 tweets, both classic ML (Random Forest) and quantum computing demonstrate significantly reduced processing times compared to the first dataset. Classic ML takes only 114.9 s (approximately 2 min) to complete, while quantum computing takes 112.7 s (approximately 1 min and 53 s). These results suggest that both techniques are highly efficient when dealing with a smaller dataset, and the difference in processing time between them is minimal.

Accuracy: Classic ML (RF) achieves a higher accuracy of 0.9241, while quantum computing achieves an accuracy of 0.9205. Classic ML maintains a slightly better accuracy in this case, but the difference remains small, indicating that both techniques perform well in accurately predicting sentiment for this dataset. Precision, Recall, and F1 Score: Both classic ML and quantum computing approaches maintain high precision, recall, and F1 scores. The precision, recall, and F1 score values for classic ML are 0.9286, 0.9241, and 0.9249, respectively. For quantum computing, the values are 0.92456, 0.9205, and 0.9214, respectively. These metrics suggest that both approaches are effective at identifying positive instances and capturing relevant sentiment information in the dataset.

## Limitations

There are certain limitations to adopting quantum computing and machine learning for Arabic language sentiment classification in social media^51,52^:

1. Limited availability of quantum computing hardware: The current state of quantum computing hardware is still in its early stages of development and is not yet widely available. This can limit the amount of data that can be used for quantum computing, and can also make it difficult to scale up the size and complexity of the sentiment analysis tasks.
2. Limited availability of quantum computing software: Quantum computing requires specialized software that is not yet widely available. This can make it difficult for researchers and developers to experiment with and implement quantum computing algorithms for sentiment analysis in Arabic language.
3. Complexity of quantum algorithms: Quantum computing algorithms for sentiment analysis can be complex and difficult to implement. This requires specialized knowledge and expertise in quantum computing, which may not be widely available.
4. Need for specialized quantum programming languages: Quantum computing requires a different programming paradigm than classical computing, and requires specialized quantum programming languages such as Qiskit or Cirq. This can make it difficult for researchers and developers who are not familiar with these languages to implement quantum algorithms for sentiment analysis.
5. Variability in dialects and expressions: Arabic language is spoken by millions of people across different regions, each with their own dialects and expressions. This variability makes it difficult to develop a single sentiment analysis model that can accurately capture the sentiment expressed in all the different forms of Arabic used in social media.
6. Complexity of Arabic grammar: Arabic language has a complex grammar that includes features such as gender, case, and tense. This complexity can make it difficult to extract sentiment information from text, particularly for quantum computing algorithms that require a simplified representation of the data.
7. Noise and errors in quantum computing: Quantum computing is susceptible to noise and errors, which can affect the accuracy and reliability of the sentiment analysis results. This requires specialized techniques such as error correction and fault tolerance, which can be difficult to implement.
8. Integration with existing systems: Sentiment analysis is often integrated with other systems such as social media platforms, customer relationship management (CRM) systems, and marketing automation systems. Integrating quantum computing and machine learning algorithms for sentiment analysis with these existing systems can be a complex and challenging task, requiring specialized knowledge and expertise.
9. Limited to two datasets of Arabic tweets—results may not generalize to other types/sizes of Arabic language datasets.
10. Only considered sentiment analysis task—performance could differ for other document classification problems.
11. Quantum computing technique used was a generic approach—tailored quantum algorithms may improve results.
12. Processing times may vary with different hardware/system specifications.

## Discussion and future work

Sentiment analysis in Arabic language social media is a challenging task due to the complexity of the Arabic language, the variability in dialects and expressions, and the lack of large, high-quality labeled datasets. Quantum computing and machine learning offer promising solutions for sentiment analysis in Arabic language social media, but there are several challenges that must be carefully considered and addressed.

The exponential growth of digital data generated by Arabic speakers has created a pressing need for effective and efficient document classification techniques. While both quantum computing and machine learning have shown promise in this field, there is a noticeable lack of research exploring their performance specifically on the Arabic language. This paper aims to address this gap by conducting a comparative study of quantum computing and machine learning techniques for Arabic document classification, utilizing two distinct datasets.

The first dataset comprises 213,465 Arabic tweets, and it serves as the basis for sentiment analysis. Both classical machine learning (ML) and quantum computing approaches demonstrate high accuracy in sentiment prediction. Quantum computing slightly outperforms classical ML, achieving an accuracy rate of approximately 82.39%, while classical ML achieves an accuracy rate of 82.15%. The computational time for quantum computing is approximately 59 min, slightly faster than the one-hour processing time of classical ML. Precision, recall, and F1 score metrics further validate the effectiveness of both approaches in predicting sentiment in Arabic tweets. Classical ML exhibits precision, recall, and F1 score values of 0.8215, 0.8175, and 0.8121, respectively, while quantum computing achieves values of 0.8239, 0.8199, and 0.8147, respectively.

Moving on to the second dataset, which consists of 44,000 tweets, both classical ML (specifically, the Random Forest algorithm) and quantum computing demonstrate significantly reduced processing times compared to the first dataset. However, there is no substantial difference in processing time between the two approaches. Classical ML completes the analysis in approximately 2 min, whereas quantum computing takes around 1 min and 53 s. In terms of accuracy, classical ML achieves a slightly higher rate of 92.41%, compared to 92.05% for quantum computing. Nevertheless, both approaches achieve high precision, recall, and F1 scores, indicating their effectiveness in accurately predicting sentiment in the dataset. Classical ML attains precision, recall, and F1 score values of 0.9286, 0.9241, and 0.9249, respectively, while quantum computing achieves values of 0.92456, 0.9205, and 0.9214, respectively.

The analysis of these metrics indicates that quantum computing approaches are particularly effective in identifying positive instances and capturing relevant sentiment information in large datasets, as demonstrated by the first dataset. On the other hand, traditional machine learning techniques exhibit faster processing times when dealing with smaller dataset sizes, as observed in the second dataset. These findings shed light on the strengths and limitations of quantum computing and machine learning for Arabic document classification. They highlight the potential of quantum computing in achieving high accuracy, particularly in scenarios where traditional machine learning techniques may face challenges.

This study contributes valuable insights to the development of more accurate and efficient document classification systems for Arabic data. By showcasing the advantages and trade-offs of both quantum computing and classical machine learning, it lays the groundwork for future research and encourages the exploration of quantum computing techniques in Arabic text analysis. Ultimately, the findings of this study have the potential to enhance the accuracy and efficiency of document classification systems for Arabic speakers, thus addressing the growing need for effective language processing tools in the digital era.

There are a list of potential areas for future work in quantum computing and machine learning for Arabic language sentiment classification in social media:

1. Developing larger, high-quality labeled datasets for sentiment analysis in Arabic language, which can be used to train and evaluate machine learning models and to test quantum computing algorithms.
2. Developing specialized quantum computing algorithms for sentiment analysis in Arabic language that can handle the complexity of Arabic grammar, the variability in dialects and expressions, and the noise and errors that are inherent in quantum computing.
3. Developing more efficient quantum computing algorithms for sentiment analysis that can handle larger data sets, and that can be implemented on current and future quantum computing hardware.
4. Integrating quantum computing algorithms with existing machine learning models and systems for sentiment analysis, to leverage the strengths of both approaches.
5. Developing new techniques for error correction and fault tolerance in quantum computing algorithms for sentiment analysis, to improve the accuracy and reliability of the results.
6. Developing new techniques for data preprocessing and feature selection in machine learning algorithms for sentiment analysis, to improve the accuracy and efficiency of the models.
7. Developing new techniques for handling bias and fairness in machine learning algorithms for sentiment analysis, to ensure that the results are fair and unbiased for all groups.
8. Developing new techniques for handling privacy and security concerns in sentiment analysis, particularly with respect to the processing of personal data.
9. Developing new techniques specifically tailored to address the challenges associated with imbalanced data.
10. Developing new techniques for handling variability in dialects and expressions in Arabic language sentiment analysis, to improve the accuracy and relevance of the results.
11. Developing new techniques for handling the complexity of Arabic grammar in sentiment analysis, to improve the accuracy and relevance of the results.
12. Developing new techniques for sentiment analysis across multiple languages, to enable cross-lingual sentiment analysis and to improve the accuracy and relevance of the results.
13. Developing new techniques for real-time sentiment analysis in social media, to enable real-time monitoring and response to changes in sentiment.
14. Developing new techniques for sentiment analysis in multimedia content, such as images and videos, to enable analysis of sentiment in non-textual content.
15. Developing new techniques for sentiment analysis in specific domains, such as politics, sports, or entertainment, to enable more targeted and relevant analysis of sentiment.
16. Developing new techniques for sentiment analysis in specific social media platforms, such as Twitter, Facebook, or Instagram, to enable more targeted and relevant analysis of sentiment.

Developing new techniques for sentiment analysis that take into account the context and cultural background of the users, to improve the accuracy and relevance of the results.

## Conclusion

Using quantum computing and machine learning offers promising solutions for Arabic language sentiment classification in social media. However, there are several challenges that must be carefully considered and addressed in the implementation of these technologies, such as the lack of large, high-quality labeled datasets, the complexity of Arabic grammar, the variability in dialects and expressions, and the need for specialized hardware and software for quantum computing. Both machine learning and quantum computing show promise for Arabic document classification, but research in this area is limited. This study comparatively evaluated the two approaches on sentiment analysis of Arabic tweets. For the larger dataset of 213K tweets, both techniques achieved high accuracy, with quantum computing performing slightly better. Quantum computing was also slightly faster. Metrics indicated both effectively predicted sentiment. On the smaller dataset of 44K tweets, processing times significantly reduced for both approaches, with no difference between them. Classic machine learning achieved slightly higher accuracy but similar metric scores to quantum computing. While traditional machine learning was faster on smaller datasets, quantum computing effectively identified positive instances and captured sentiment information in large datasets. The findings provide insights into the strengths and limitations of each technique. Quantum computing demonstrates high accuracy even with difficulties in traditional machine learning. This research contributes to more accurate and efficient systems for classifying Arabic data.

## Data Availability

The dataset used in this study is public and all test data are available at this portal (https://www.kaggle.com/datasets/mtesta010/arabic-asthma-tweets).
